# Two new species of the primitively segmented spider genus *Liphistius* Schiödte, 1849 (Mesothelae, Liphistiidae) from Myanmar

**DOI:** 10.3897/zookeys.882.38811

**Published:** 2019-10-23

**Authors:** Khin Pyae Pyae Aung, Xin Xu, Wai Wai Lwin, Men Zing Sang, Long Yu, Hao Liu, Fengxiang Liu, Daiqin Li

**Affiliations:** 1 Department of Zoology, University of Yangon, Kamayut Township, Pyay Road, Yangon, 11041, Myanmar; 2 Department of Biology, Taungoo Education College, Taungoo, 08101, Myanmar; 3 College of Life Sciences, Hunan Normal University, 36 Lushan Road, Changsha 410081, Hunan Province, China; 4 State Key Laboratory of Biocatalysis and Enzyme Engineering, and Centre for Behavioural Ecology and Evolution (CBEE), School of Life Sciences, Hubei University, 368 Youyi Road, Wuhan 430062, Hubei Province, China; 5 Department of Biological Sciences, National University of Singapore, 14 Science Drive 4, 117543, Singapore

**Keywords:** *
Liphistius
*, Myanmar, taxonomy, trapdoor spiders

## Abstract

Two *Liphistius* species of the primitively segmented spider family Liphistiidae, collected from Loikaw (Kayah State) and Pinlaung (Shan State), Myanmar, are diagnosed and described as new to science based on their genital morphology: *Liphistius hpruso***sp. nov.** (♀), *Liphistius pinlaung***sp. nov.** (♂♀).

## Introduction

**Figure 1.**
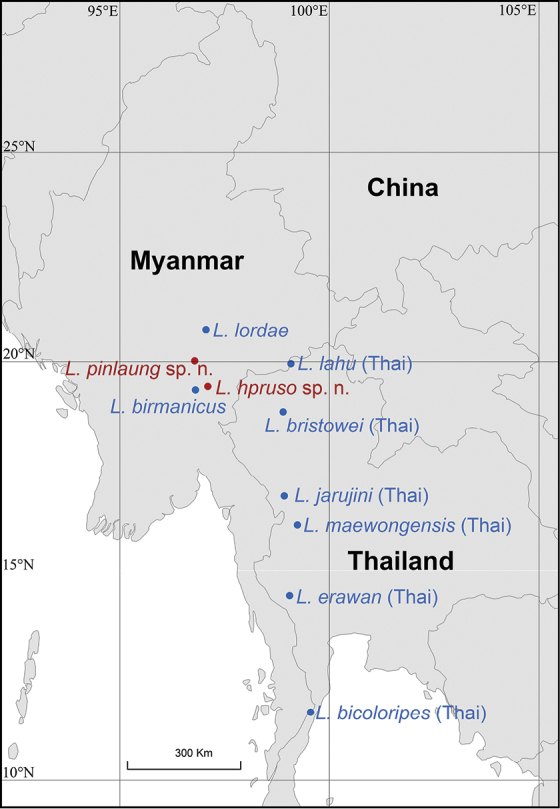
A map showing the type localities of ten *Liphistius* species in Myanmar and Thailand. Two new species are indicated in red solid circles, and two known species in Myanmar and six known species in Thailand are indicated in blue solid circles.

The segmented trapdoor spiders of the family Liphistiidae, the sister lineage to all other extant spiders, are at a pivotal position on the arachnid tree of life (Platnick and Gertsch 1976; Xu et al. 2015a). Liphistiids are often regarded as ‘living fossils’ (Bristowe 1975) since they retain many plesiomorphic characters such as the presence of abdominal tergal plates and the position of the spinnerets on the median area of the opisthosoma (Pocock 1892; Platnick and Gertsch 1976; Haupt 1983, 2003; Coddington and Levi 1991). Two allopatric subfamilies, Liphistiinae Thorell, 1869 and Heptathelinae Kishida, 1923, are distributed in East (China, Japan and Vietnam) and South-east (Laos, Malaysia, Myanmar, Indonesia (Sumatra), and Thailand) Asia, respectively (Xu et al. 2015a, b; World Spider Catalog 2019). Liphistiinae contains 55 described species in the single genus, *Liphistius* Schiödte, 1849: 33 species from Thailand, 16 from peninsular Malaysia, one from both Thailand and peninsular Malaysia, two from Myanmar, one from Laos, one from Indonesia (Sumatra), and one from both Laos and Thailand (World Spider Catalog 2019). Surprisingly, only two species, *L. birmanicus* Thorell, 1897 and *L. lordae* Platnick & Sedgwick, 1984, have been reported from Myanmar since the first species was described in 1897 (Thorell 1897; Platnick and Sedgwick 1984; Schwendinger 1990; Xu et al. 2015b), given that its landmass is even larger than Thailand, its climate and geological topography are similar to those of Thailand, and it shares the mountain ranges with Thailand across a 10° latitude range (Fig. 1). Since at least six species in Thailand (*L. albipes* Schwendinger, 1995, *L. bristowei* Platnick & Sedgwick, 1984, *L. erawan* Schwendinger, 1996, *L. jarujini* Ono, 1988, *L. lahu* Schwendinger, 1998, and *L. maewongensis* Sivayyapram et al., 2017) occur very close to its border with Myanmar, one would expect a comparable species diversity also in Myanmar (Fig. 1).

To document species diversity of *Liphistius* in Myanmar, we carried out two expeditions in East Myanmar in 2018. In this study, we report two new species of *Liphistius* after having examined the specimens collected from our expeditions in 2018.

## Materials and methods

### Specimen acquisition

**Figure 2.**
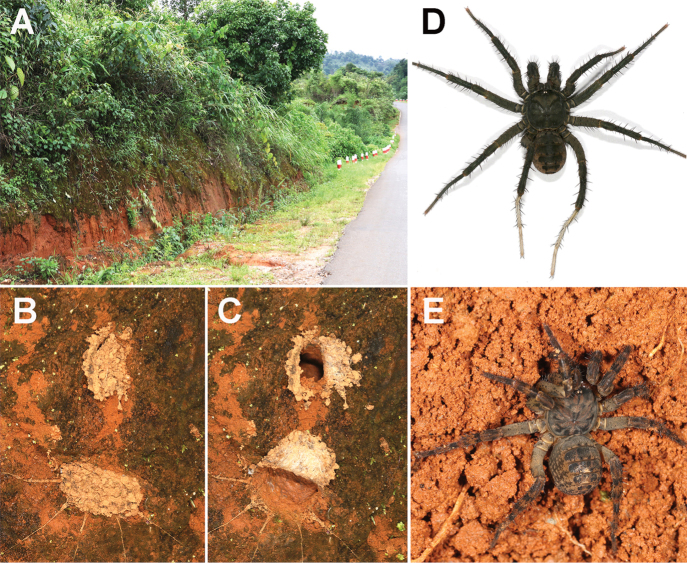
Macrohabitat, burrow with trapdoors, and general somatic morphology (taken in the field) of *Liphistius pinlaung* sp. nov. **A** macrohabitat **B** a burrow with two trapdoors closed **C** a burrow with two trapdoors opened **D** male (XUX-2018-164, holotype) **E** female (XUX-2018-162).

All specimens were collected from Loikaw (Kayah State) and Pinlaung (Shan State), Myanmar (Figs 1, 2). They were collected alive and fixed in absolute ethanol if they were adults, and then their right four legs were removed to be stored at −80 °C for molecular work. The rest of each specimen was preserved in 80% ethanol as the voucher for morphological examination.

### Morphological examination

Specimens were examined using an Olympic SZX16 Leica stereomicroscope. Genitalia were cleared in boiling KOH for a few minutes to dissolve soft tissues, examined and photographed with an Olympic BX53 or SZX7 compound microscope and a Canon 7D camera. All voucher specimens are deposited at the Centre for Behavioural Ecology and Evolution (**CBEE**), College of Life Sciences, Hubei University, Wuhan, Hubei Province, China. Genital anatomical terminology follows Schwendinger and Ono (2011) and Schwendinger (2017). All measurements were carried out under a Leica M205 digital microscope and are given in millimetres. Leg and palp measurements are given in the following order: total leg length (femur + patella + tibia + metatarsus + tarsus), total palp length (femur + patella + tibia + tarsus).

Abbreviations used in the text:

**ALE** anterior lateral eye;

**AME** anterior median eye;

**CDO** central dorsal opening;

**CT** contrategulum;

**E** embolus;

**GA** genital atrium;

**PC** paracymbium;

**PeP** paraembolic plate;

**PLE** posterior lateral eye;

**PME** posterior median eye;

**PPl** poreplate;

**PS** posterior stalk;

**RC** receptacular cluster;

**ST** subtegulum;

**T** tegulum;

**TiA** tibial apophysis.

## Taxonomy

### Family Liphistiidae Thorell, 1869

#### Subfamily Liphistiinae Thorell, 1869

##### Genus Liphistius

Taxon classificationAnimaliaAraneaeLiphistiidae

Schiödte, 1849

5EC4AF30-4502-5364-BA83-DB6555045B46

###### Type species.

*Liphistius desultor* Schiödte, 1849

###### Diagnosis.

**Figure 3.**
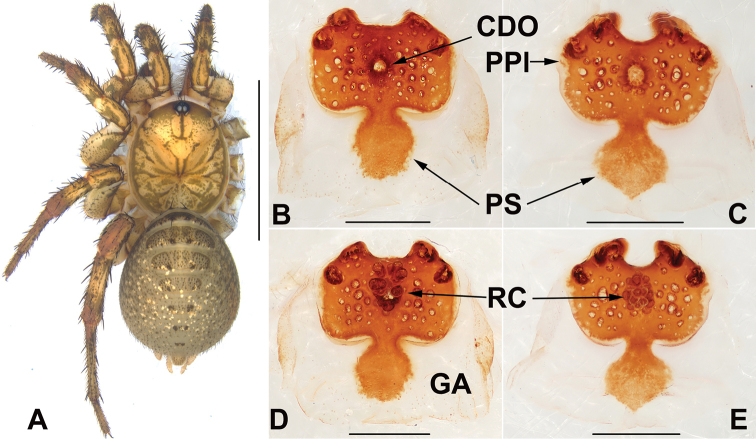
General somatic morphology (taken after fixed by ethanol) and female genitalia of *Liphistius hpruso* sp. nov. **A** female (XUX-2018-151, holotype) **B, D** XUX-2018-151 **C, E** XUX-2018-152 **B, C** vulvae, dorsal view **D, E** vulvae, ventral view. Scale bars: 10 mm (**A**); 0.5 mm (**B–E**).

**Figure 4.**
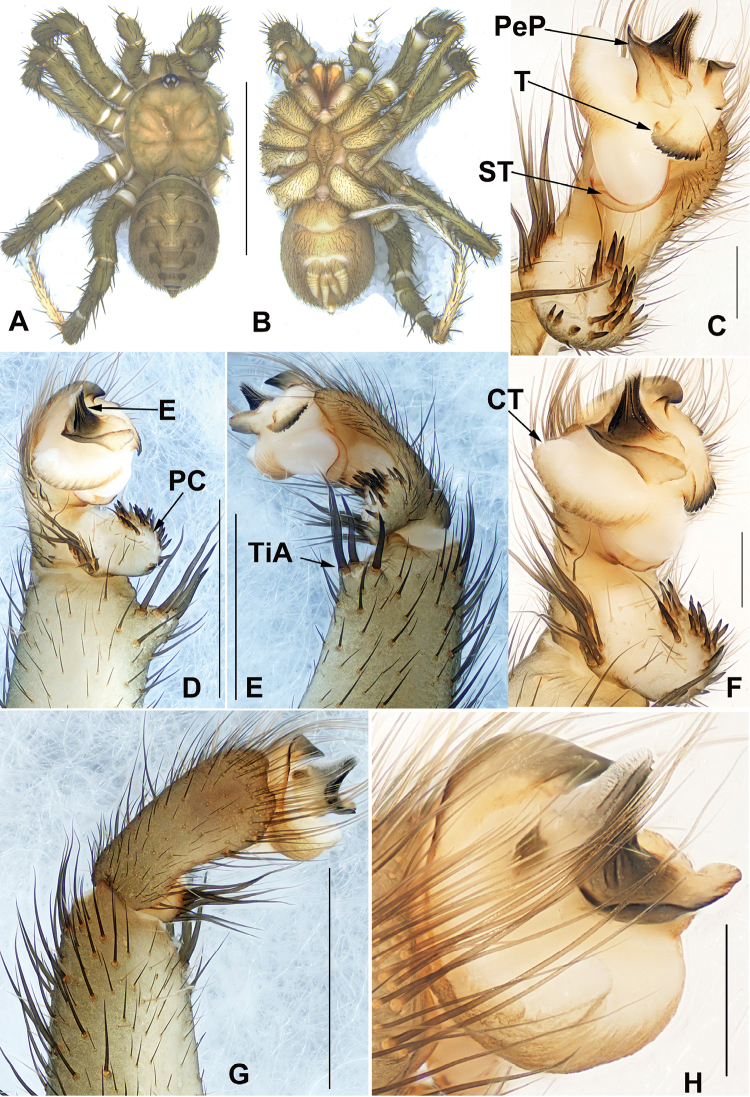
General somatic morphology (taken after fixed by ethanol) and male palp of *Liphistius pinlaung* sp. nov. (XUX-2018-164, holotype) **A, B** male: **A** dorsal view **B** ventral view **C, F, H** palp distal view **D** palp ventral view **E** palp retrolateral view **G** palp prolateral view. Scale bars: 10 mm (**A, B**); 2 mm (**D, E, G**); 0.5 mm (**C, F, H**).

**Figure 5.**
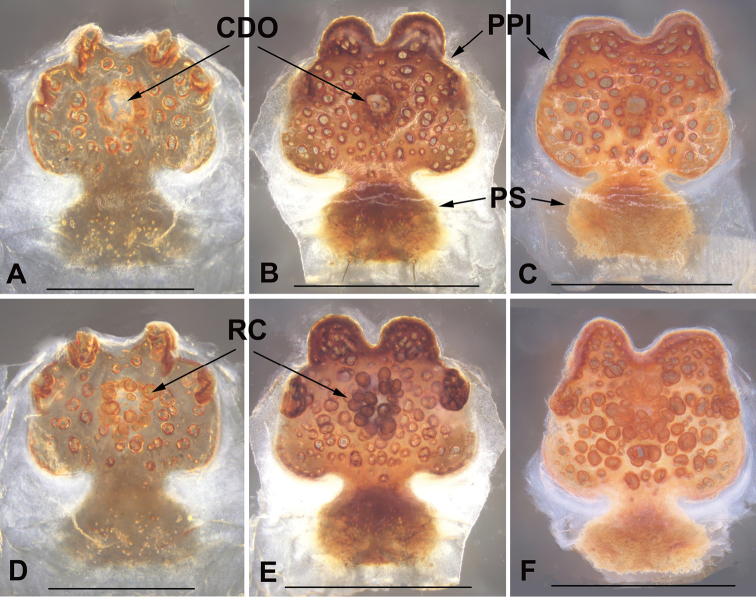
Female genitalia of *Liphistius pinlaung* sp. nov. **A, D** XUX-2018-167 **B, E** XUX-2018-169A **C, F** XUX-2018-169J **A–C** vulvae, dorsal view **D–F** vulvae, ventral view. Scale bars: 0.5 mm (**A, D**); 1 mm (**B, C, E, F**).

*Liphistius* can be distinguished from all other liphistiid genera by the male palp that possesses a tibial apophysis (Fig. 4D, E), and by the presence of a poreplate and a median receptacular cluster in female genitalia (Figs 3B–E, 5A–F).

###### Distribution.

Laos, Malaysia, Myanmar, Indonesia (Sumatra) and Thailand.

##### Liphistius hpruso sp. nov.

Taxon classificationAnimaliaAraneaeLiphistiidae

C6A574C4-8754-5050-BC88-D7890A8C2C3B

http://zoobank.org/DC7346A9-F429-4197-A207-7747C24EC9E7

Fig. 3

###### Type material.

***Holotype***: MYNAMAR · ♀; Kayah State, Loi Kaw District, Hpruso, Dokhule, along a small road near Queen of Peace Church; 19.41N, 97.10E; alt. 1157 m; 17 July 2018; D. Li, F.X. Liu, X. Xu and L. Yu leg.; XUX-2018-151. Deposited in CBEE.

***Paratype***: MYANMAR · 1 ♀; same data as for holotype; XUX-2018-152. Deposited in CBEE.

###### Diagnosis.

Females of *Liphistius hpruso* sp. nov. resemble those of *L. birmanicus* and *L. pinlaung* sp. nov. by the poreplate with paired anterior lobes and anterolateral lobes, but can be distinguished from those of *L. birmanicus* and *L. pinlaung* sp. nov. by the globosely receptacular cluster (Fig. 3D, E), and the smaller anterolateral lobes of the pore plate (Fig. 3D, E); from *L. pinlaung* sp. nov. by the narrower posterior stalk; from the other *Liphistius* species by the pore plate with similarly sized anterior lobes and anterolateral lobes, and with the narrow posterior stalk (Fig. 3B–E).

###### Description.

**Female** (holotype). Total length, excluding chelicerae, 16.85. Four thick setae on clypeus (Fig. 3A). Carapace 7.02 long, 6.16 wide, longer than wide, light brown, furnished with few short, scattered bristles. Eight eyes on darkened ocular tubercle, ALE > PLE > PME > AME. Eye sizes and interdistances: AME 0.05, ALE 0.57, PME 0.35, PLE 0.45; AME-AME 0.09, AME-ALE 0.17, PME-PME 0.08, PME-PLE 0.13, ALE-PLE 0.17, ALE-ALE 0.19, PLE-PLE 0.41, AME-PME 0.09. Chelicerae light and glabrous proximally, robust, dark brown; promargin of chelicerae groove with ten denticles of variable size. Labium 0.77 long, 1.47 wide. Sternum 3.61 long, 1.83 wide, brown with several setae. Opisthosoma 9.50 long, 7.53 wide, dark brown, with 12 tergites, and eight spinnerets. Legs brown with strong hairs and spines, long and short black sparse setae, with three tarsal claws. Measurements: palp 10.59 (3.18 + 2.20 + 2.69 + 2.52), leg I 11.77 (3.09 + 2.31 + 2.85 + 1.99 + 1.52), leg II 12.17 (2.72 + 2.21 + 2.92 + 2.49 + 1.83), leg III 12.45 (2.80 + 2.22 + 3.16 + 2.70 + 1.57), leg IV 20.99 (4.87 + 2.79 + 4.31 + 5.96 + 3.06).

Female genitalia: vulva with nearly rectangular pore plate; pore plate with similarly sized anterior lobes and anterolateral lobes; distinct transition between the pore plate and posterior stalk (Fig. 3B–E); posterior stalk narrow and long; receptacular cluster spherical and small; central dorsal opening small and circular (Fig. 3B–E).

**Male.** unknown.

###### Entomology.

“hpruso” refers to the type locality of this species.

###### Distribution.

Myanmar (Loi Kaw District, Kayah State).

##### Liphistius pinlaung sp. nov.

Taxon classificationAnimaliaAraneaeLiphistiidae

0EE6BC3F-A326-5330-9E4C-1EFFB0E36051

http://zoobank.org/1E893A2D-D43C-4B16-A19D-77352D7EE823

Figs 4 , 5

###### Type material.

***Holotype***: MYNAMAR · ♂; Shan State, Pinlaung Township, ca.14 km to Pinlaung from Pekon; 20.02N, 96.79E; alt. 1410 m; 19 July 2018; D. Li, F.X. Liu, X. Xu and L. Yu leg.; XUX-2018-164. Deposited in CBEE.

***Paratype***: MYNAMAR · 1 ♂, 5 ♀♀; same data as for holotype; XUX-2018-162, 167, 169, 169A, 169B, 169J; 19 July 2018. All specimens deposited in CBEE.

###### Diagnosis.

Males of *L. pinlaung* sp. nov. resemble those of *L. birmanicus*, *L. lordae* and *L. lahu* by the wide paraembolic plate, but can be distinguished from *L. birmanicus* by the lack of lateral process of paracymbium and by the cumulus with longer and stouter setae (Fig. 4C, D); from *L. lordae* by the wider tibial apophysis at base (Fig. 4D) and the tegulum with a dentated margin (Fig. 4C, F); from *L. lahu* by the narrower tegulum (Fig. 4C, F) and smaller paracybium (Fig. 4D, E). Females of *L. pinlaung* sp. nov. resemble those of *L. birmanicus* and *L. hpruso* sp. nov. by the poreplate with two pair of lobes, but can be distinguished from *L. birmanicus* by the wider posterior stalk, and sphere-shaped receptacular cluster (Fig. 5D–F); from *L. hpruso* sp. nov. by the wider posterior stalk and larger anterior lobes of the poreplate (Fig. 5A–F); from the other *Liphistius* by the poreplate with four anterior lobes (Fig. 5D–F).

###### Description.

**Male** (holotype). Total length, excluding chelicerae, 12.71. Carapace 5.86 long and 5.47 wide, longer than wide, olive-green due to being fixed in ethanol immediately after molting, furnished with few short, scattered bristles (Fig. 4A). ALE>PLE>PME>AME, eye sizes and interdistances: AME 0.05, ALE 0.55, PME 0.31, PLE 0.48, AME-AME 0.10, AME-ALE 0.07, PME-PME 0.09, PME-PLE 0.09, ALE-PLE 0.09, ALE-ALE 0.11, PLE-PLE 0.38, AME-PME 0.09. Chelicerae robust, promargin of chelicerae groove with ten strong denticles of variable size. Labium 0.86 long and 0.89 wide, wider than long, fused with sternum and slightly pale olive-green (Fig. 4B). Sternum 2.94 long and 1.05 wide, longer than wide, and a few weakly spined setae on the anterior tip and many long spined setae on the posterior tip, elongated posterior tip (Fig. 4B). Opisthosoma 7.17 long and 4.92 wide, with 12 tergites, the fifth largest, eight spinnerets (Fig. 4B). Legs with strong hairs and spines. Measurements: leg I 16.99 (4.32 + 2.55 + 3.55 + 4.66 + 1.92), leg II 18.06 (4.32 + 2.41 + 3.74 + 5.18 + 2.41), leg III 18.46 (4.44 + 1.85 + 2.83 + 6.68 + 2.66), leg IV 20.40 (3.56 + 1.52 + 4.25 + 8.46 + 2.63).

***Palp***: Tibial apophysis with four long spines of different lengths (Fig. 4D, E), paracymbium large and wide, many setae situated at the tip and a row of several tapering spines one the indistinct cumulus (Fig. 4C, D, F); subtegular apophysis weakly developed (Fig. 4C); contrategulum with conical, tip blunt with a short process (Fig. 4C, F), distal edge widely arched, with a smooth and sharp edge (Fig. 4F–H); tegulum small and the terminal apophysis with finely dentated margin (Fig. 4C, E, F); paraembolic plate short, widely rounded, embolic parts adjacent (Fig. 4D, F, H); embolus long and conical, basally sclerotized, with 3–4 longitudinal ridges that reach to tip (Fig. 4C, D, F).

**Female.** Total length, excluding chelicerae, 14.46. Carapace 6.70 long, 6.07 wide, light brown, furnished with few short, scattered bristles. Four thick setae on clypeus. Eight eyes on darkened ocular tubercle, ALE > PLE > PME > AME, eye size and interdistances: AME 0.09, ALE 0.61, PME 0.33, PLE 0.47, AME-AME 0.11, AME-ALE 0.16, PME-PME 0.13, PME-PLE 0.13, ALE-PLE 0.14, ALE-ALE 0.14, PLE-PLE 0.43, AME-PME 0.14. Chelicerae proximally glabrous, robust, dark brown; promargin of chelicerae groove with 14 strong denticles of variable size. Labium 0.75 long, 1.19 wide, slightly pale brown. Sternum 3.25 long, 1.59 wide, brown and weakly spined, a few setae on the outside of this area, elongated posterior tip. Opisthosoma 8.20 long, 5.73 wide, dark brown, with 12 tergites, the fifth largest, and eight spinnerets. Legs brown with strong hairs and spines, long and short black sparse setae, legs each with three tarsal claws. Measurements: palp 8.59 (2.01 + 1.67 + 2.65 + 2.27), leg I 11.75 (3.39 + 1.99 + 3.03 + 2.01 + 1.33), leg II 12.02 (2.69 + 2.05 + 3.14 + 2.45 + 1.68), leg III 13.47 (4.19 + 1.22 + 3.51 + 2.49 + 2.05), leg IV 22.4 (6.47 + 2.58 + 4.38 + 5.82 + 3.15).

***Female genitalia***: pore plate with a pair of large anterior lobes and a pair of small, strongly elevated anterolateral lobes, and anterior lobes larger than anterolateral lobes (Fig. 5D–F); distinct transition between the pore plate and posterior stalk (Fig. 5A–F); posterior stalk wide; receptacular cluster spherical and small; central dorsal opening small and circular (Fig. 5A–C).

###### Entomology.

“pinlaung” refers to the type locality of this species.

###### Distribution.

Myanmar (Pinlaung Township, Shan State).

###### Variation.

Body measurements, see Table 1. The examined female genitalia differ from each other; for the specimen of XUX-2018-169A, the central part of anterior and anterolateral lobes of the pore plate are depressed in the dorsal view (Fig. 5B), whereas the depression is absent in the other two specimens (XUX-2018-167 and 169J); the shape and size of anterior and anterolateral lobes of the pore plate, as well as the shape of anterior margin of the pore plate are rather variable (Fig. 5A–F). The size of the receptacular cluster is also slightly different (Fig. 5D–F).

###### Relationships.

*Liphistius hpruso* sp. nov. and *L. pinlaung* sp. nov. belong to the *birmanicus*-group that currently contains *L. birmanicus*, *L. lordae* and *L. lahu* based on morphological characters (Schwendinger, 1998). The two new species are closer to *L. birmanicus* than to *L. lordae* and *L. lahu* since their female poreplates possess four anterior lobes (Figs 3B–E; 5D–F).

**Table 1.** Body measurements (mm) of one male (♂) and five females (♀) of *Liphistius pinlaung* sp. nov.

**Sample number**	**Carapace**		**Opisthosoma**		**Sternum**		**labium**		**Body length**
	**length**	**width**	**length**	**width**	**length**	**width**	**length**	**width**	
XUX-2018-162 (♀)	6.70	6.08	8.20	5.73	3.25	1.57	0.75	1.19	14.46
XUX-2018-167 (♀)	5.23	4.86	4.96	3.36	2.60	1.28	0.61	0.95	10.27
XUX-2018-169 (♂)	6.54	6.34	7.06	5.34	2.84	1.08	0.59	0.94	13.58
XUX-2018-169A (♀)	7.05	5.99	7.43	5.53	3.29	1.57	0.91	1.38	14.47
XUX-2018-169B (♀)	7.62	6.55	7.50	5.24	3.10	1.48	0.89	1.37	14.49
XUX-2018-169J (♀)	7.47	6.66	7.05	4.96	3.75	1.65	0.72	1.59	14.09

## Supplementary Material

XML Treatment for Liphistius

XML Treatment for Liphistius hpruso

XML Treatment for Liphistius pinlaung
